# Increased Functional Coupling between VTA and Hippocampus during Rest in First-Episode Psychosis

**DOI:** 10.1523/ENEURO.0375-20.2021

**Published:** 2021-03-17

**Authors:** David F. Gregory, Jane M. Rothrock, Maria Jalbrzikowski, William Foran, David F. Montez, Beatriz Luna, Vishnu P. Murty

**Affiliations:** 1 Department of Psychology, Temple University, Philadelphia, PA 19122; 2 Department of Psychiatry, University of Pittsburgh, Pittsburgh, PA 15213; 3 Department of Neurology, Washington University in St. Louis, St. Louis, MO 63110

**Keywords:** hippocampus, psychosis, resting state fMRI, striatum, ventral tegmental area

## Abstract

Animal models suggest that interactions between the hippocampus and ventral tegmental area (VTA) underlie the onset and etiology of psychosis. While a large body of research has separately characterized alterations in hippocampal and VTA function in psychosis, alterations across the VTA and hippocampus have not been characterized in first-episode psychosis (FEP). As the phase of psychosis most proximal to conversion, studies specifically focused on FEP are valuable to psychosis research. Here, we characterize alterations in VTA-hippocampal interactions across male and female human participants experiencing their first episode of psychosis using resting state functional magnetic resonance imaging (rsfMRI). In comparison to age and sex matched healthy controls (HCs), FEP individuals had significantly greater VTA-hippocampal functional coupling but significantly less VTA-striatal functional coupling. Further, increased VTA-hippocampal functional coupling in FEP correlated with individual differences in psychosis-related symptoms. Together, these findings demonstrate alterations in mesolimbic-hippocampal circuits in FEP and extend prominent animal models of psychosis.

## Significance Statement

We characterized differences in functional coupling between the VTA and hippocampus in FEP. We found enhanced functional coupling between the VTA and right hippocampus in FEP, which correlated with individual differences in psychosis symptoms. We broaden a growing literature characterizing mesolimbic-hippocampal interactions, showing a prominent role for this circuit in psychosis. Further, our findings connect well with animal models of psychosis which detail alterations in this circuit as a risk factor for developing psychosis. These findings support a model of increased functional interactions between the hippocampus and VTA with etiology of psychosis and may predict individual differences in psychosis-related symptoms.

## Introduction

Psychosis is associated with alterations in the mesolimbic dopamine system, which has been linked with psychosis onset and related positive symptoms (i.e., delusions, paranoia). Rodent studies modeling mesolimbic deficits in psychosis show that hyperactivity in baseline hippocampal function results in an upregulation of tonic dopamine signaling in the ventral tegmental area (VTA; Lisman and Grace, 2005). In the methylazoxymethanol acetate (MAM) model, a rodent model of psychosis, reduced GABA-ergic signaling in the hippocampus leads to hippocampal hyperactivity, leading to an increased tonic signaling within the VTA (Lodge and Grace, 2011). A growing body of research has shown that mesolimbic-hippocampal interactions in healthy human adults are homologous to animal models (Miendlarzewska et al., 2016; Murty and Alison Adcock, 2017), such that there is functional coupling between the VTA and hippocampus during novelty and reward processing (Miendlarzewska et al., 2016; Murty and Alison Adcock, 2017) and during resting state (Kahn and Shohamy, 2013; Gregory et al., 2020). In the current study, we used resting state functional magnetic resonance imaging (rsfMRI) to compare functional coupling of the mesolimbic system and hippocampus between cohorts of individuals in their first-episode of psychosis (FEP) and healthy controls (HCs).

Current research in FEP, described as the first time an individual receive a diagnosis of psychosis/are within their first episode of psychosis, has been sparce in the psychosis literature (Bommersbach et al., 2021). However, characterizing FEP individuals is important to understand the onset and progression of this disorder. The clinical expression of psychosis varies between individuals at different stages of disorder progression, thus understanding mesolimbic dysfunction in FEP provides an opportunity to detail markers of psychosis closer to the onset of the disorder in the absence of confounds of prolonged medication and chronic illness (Breitborde et al., 2015).

Previous research has shown disruptions in task-evoked VTA activation in individuals with psychosis (Ermakova et al., 2018; McCutcheon et al., 2019). Increased dopaminergic signaling in prodromal populations reflects negative symptoms and predicts conversion to psychosis (Howes et al., 2009; Abi-Dargham, 2017). In parallel, individuals with psychosis reliably show reductions in hippocampal structural integrity (van Erp et al., 2016; Vargas et al., 2018), impairments in behavioral indices of hippocampal function (Saykin, 1991; Heinrichs and Zakzanis, 1998; Lee and Park, 2005), increases in hippocampal glutamate levels measured by spectroscopy (Bossong et al., 2019; Bustillo et al., 2019), and increases in cerebral blood flow in hippocampus (Allen et al., 2018). Last, hippocampal and VTA deficits are present across the phases of psychotic illness and in those at high risk for developing the disorder (Kraguljac et al., 2013; Baglivo et al., 2018; Modinos et al., 2018).

Despite animal models of psychosis predicting VTA modulation of medial temporal lobe (MTL) through alterations which impact mesolimbic-hippocampal interactions (Lodge and Grace, 2008; Gomes and Grace, 2018), VTA and hippocampal activation are often characterized separately in patient populations. Recently, research has begun to characterize interactions across the VTA and hippocampus in psychosis-related populations (for review, see Modinos et al., 2015). A study found that, during the processing of novel environments, individuals at clinical high risk (CHR) for developing psychosis exhibited attenuated functional coupling between the VTA and hippocampus; moreover, these alterations were greater in individuals that converted to having psychosis versus not (Modinos et al., 2020). Further, hippocampus-VTA connectivity was altered in a sample of individuals with chronic schizophrenia, finding a correlation with positive symptoms during resting state but importantly Nakamura and colleagues did not find differences across individuals with schizophrenia and controls as well as those with other neuropsychiatric disorders (Nakamura et al., 2020). Overall, these studies highlight a critical role for hippocampus-VTA circuits in psychosis, which has been found within animal models but open questions remain regarding the characterization of this circuit in human research on FEP.

In the current study, we assessed group differences in rsfMRI functional coupling in FEP versus HC (FEP: *N* = 46; HC: *N* = 32), with purpose to characterize how deficits in mesolimbic-hippocampal interactions are related to psychosis symptoms. Here, we classify FEP as individuals who are in a first episode of psychosis which is proximal to the onset of the disorder (Manivannan et al., 2019), which can extend over prolonged periods of time if the episode is continuous (i.e., months). Our hypothesis was that the FEP group would display greater functional coupling between the VTA and hippocampus compared with HC. In line with animal models, we also hypothesize that these rsfMRI functional coupling differences would be specific to the hippocampus and not appear in other regions of the MTL [i.e., perirhinal cortex (PRC)] or other targets in the mesolimbic network [i.e., nucleus accumbens (NAcc)].

## Materials and Methods

### Participants

Participants were recruited from the outpatient services of the Western Psychiatric Institute and Clinic of University of Pittsburgh Medical Center and extensively evaluated using medical, neurologic, and psychiatric assessments. The final sample (from a total of 80 participants, two removed for excessive head motion >2.5 mm) included 46 FEP [M_age_(SD) = 22.4(4.7); 15 females; number of individuals on anti-psychotics = 32] and 32 HC [M_age_(SD) = 21.4(3.4); 11 females; number of individuals on anti-psychotics = 0]. All participants or their legal guardians provided written informed consent or assent according to the guidelines of the University of Pittsburgh Institutional Review Board after study procedures were fully explained, and were compensated for their participation on completion of the study. Data were collected as part of a larger study on first-episode psychosis (FEP; for which author B.L. can be contacted for data requests).

Exclusion criteria for all participants included: significant neurologic disorder, head injury, or medical illness affecting the central nervous system function, IQ lower than 75 (determined using the Wechsler Abbreviated Scale of Intelligence), DSM-IV substance dependence or substance abuse disorder within the prior six months, or any contraindications for use of MRI. Individuals were included in the FEP group if they were within their first episode of psychotic symptoms and were antipsychotic-naive or prescribed antipsychotic treatment across their lifetime for less than two months. Diagnoses were determined using all available clinical information and data gathered from a Structured Clinical Interview for DSM-IV (SCID) conducted by a trained Master’s-level or PhD-level clinician. Information on SCID in use of FEP diagnosis followed methods described previously (Manivannan et al., 2019). At least three senior clinical researchers would then arrive at a consensus regarding diagnosis at a diagnostic conference in which all available clinical data were reviewed. Illness duration for each patient was also determined in the consensus conference after a review of historical information about psychosis onset. Individuals were only included in the HC group if they had no lifetime history of a major psychiatric disorder or antipsychotic treatment, as well as no first-degree family member with a history of a psychotic disorder.

### Data acquisition

All participants completed a rsfMRI scan as part of a larger protocol that included structural imaging, diffusion imaging, and task-based fMRI. For the current study, only rsfMRI data were analyzed, which was collected ∼35 min into the scan session after structural and task fMRI (Jalbrzikowski et al., 2018). MRI data were collected on a 3.0 T Siemens Tim Trio at the University of Pittsburgh Medical Center Magnetic Resonance Center using a 32-channel phase array head coil. For fMRI acquisition, a single-shot echo-planar imaging sequence sensitive to BOLD contrast (T2*) was collected (TR = 1000 s; TE = 30 ms; flip angle = 55°; voxel size: 2.3-mm isotropic voxels AC/PC aligned, 60 contiguous axial slices; 300 acquisitions). We also collected a high-resolution anatomic image (MPRAGE, voxel size: 1.0-mm isotropic voxels). We preprocessed rsfMRI scans using an established pipeline (Hallquist et al., 2013). Briefly, the preprocessing steps were: (1) simultaneous 4D slice-timing and head motion correction, (2) wavelet de-spiking, (3) nonlinear warping to MNI space, (4) spatial smoothing with a 5-mm Gaussian kernel, (5) and simultaneous bandpass filtering (0.009–0.08 Hz) and nuisance regression. For nuisance regression, we also regressed out effects of head motion (6° of translation/rotation and their first derivative) and non-gray matter signal (i.e., white matter and cerebrospinal fluid, and their first derivative). Given that head motion is critical for between group comparisons, we additionally calculated two quality control measures of motion: volume-to-volume framewise displacement (FD) and the RMS derivative of fMRI time-series (DVARS). For each participant, we identified volumes that had an FD > 0.3 mm and/or DVARS > 20 (computed after wavelet de-spiking). These “flagged” volumes were excluded for all pair-wise correlations (detailed below). Resting-state scans consisted of 360 acquisitions with a TR of 1260 ms/scan, yielding a total scan duration of 7.5 min during which participants kept their eyes open and focused on a fixation cross for the duration of the scan.

### Analysis

**Figure 1.**
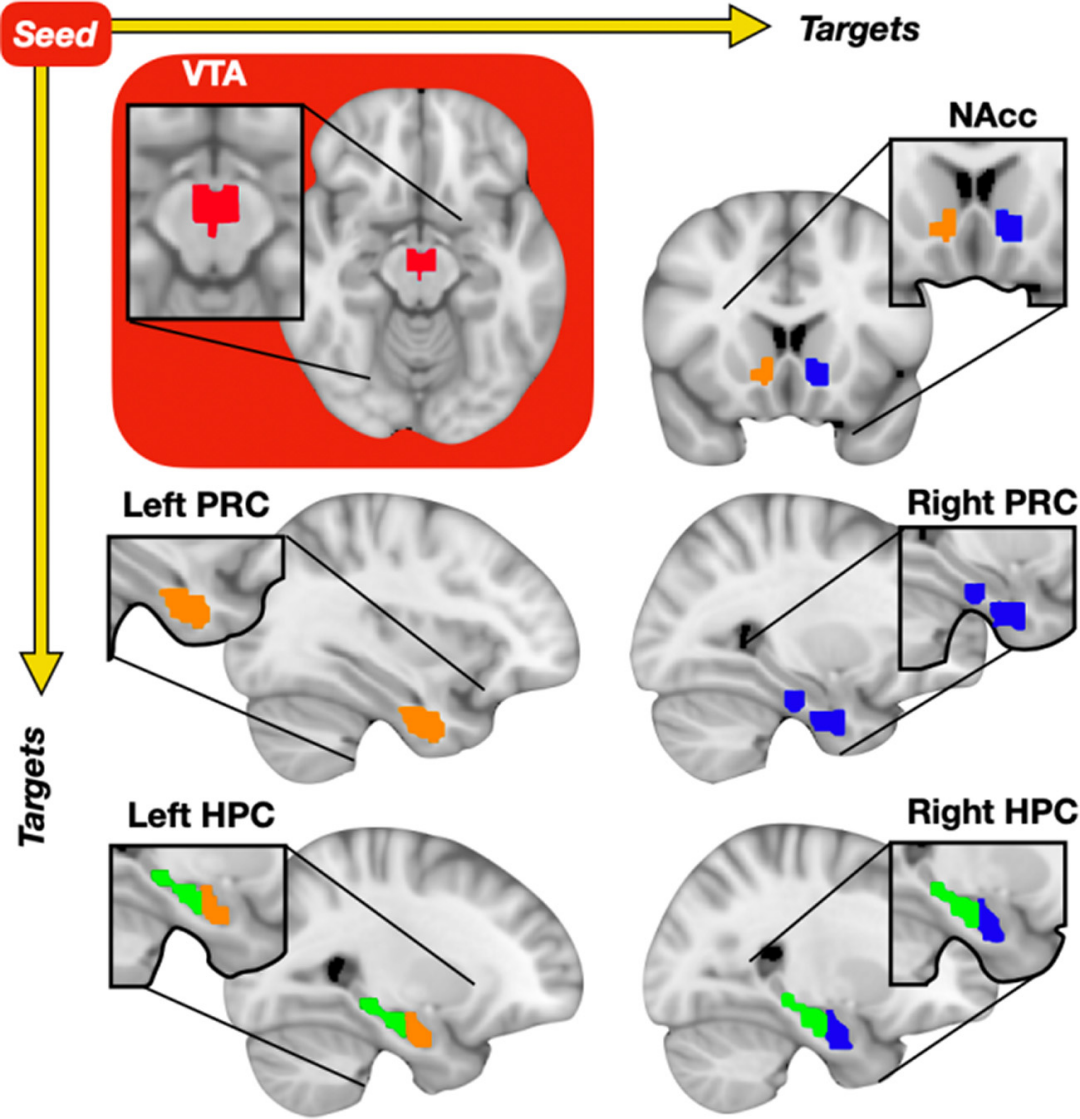
Lateral ROI diagram. Axis depicts analysis of ROI seed to each ROI target separately. ROI seed (in red box): VTA (red). ROI targets (white background): NAcc (top right; pink, light green), PRC (middle; purple, light blue), and anterior hippocampus (HPC; bottom; orange, dark blue). For visualization purposes remaining left and right HPC ROI depicted in dark green.

For each individual participant, time-series were extracted from our seven regions of interest (ROIs). All ROIs were defined from probabilistic atlases and thresholded at 75%. The hippocampus and NAcc were defined from the Harvard-Oxford Subcortical Atlas (https://neurovault.org/collections/262/). We limited our analysis to the anterior third of the long-axis of the hippocampus, given the established role of this region in psychosis (Schobel et al., 2009; McHugo et al., 2019) as well as in mesolimbic-hippocampal interactions (Murty et al., 2017). The VTA was defined from a probabilistic atlas defined in a prior resting state study (Murty et al., 2014), and the PRC was defined from a probabilistic atlas defined in an emotional memory study (Ritchey et al., 2019). Figure 1 displays the ROIs used for analyses. Next, we extracted time series for each ROI and generated pair-wise correlations removing flagged volumes using the *corr* function in MATLAB. All correlation *R* values were converted to Z-scores using a Fischer transform. All flagged time points were removed from time-series analysis. Our protocol implements the removal of participants with more than a quarter of time points flagged for exclusion, but no participants met this threshold. Then, group-level analyses were performed using the *t.test* function in R, with pair-wise correlation scores of functional coupling between ROI seed (the VTA) to each ROI target (i.e., right anterior hippocampus) as the dependent variable and cohort (FEP or HC) as the independent variable. All significant analyses were confirmed to remain significant when including age and sex as additional regressors using the lm function in R. These analyses were corrected for multiple comparisons using Bonferroni correction. Finally, within the FEP group, we ran simple regressions between functional coupling scores for the VTA-hippocampus with symptom scores on the Brief Psychiatric Rating Scale. We then verified in *post hoc* regression analyses using the *partialcorr* function in MATLAB with age, sex, and antipsychotic status covariates. Finally, we also included functional coupling between the VTA-NAcc to determine the specificity of our brain-behavior correlations by including it as another covariate using the *partialcorr* function in MATLAB.

## Results

**Figure 2.**
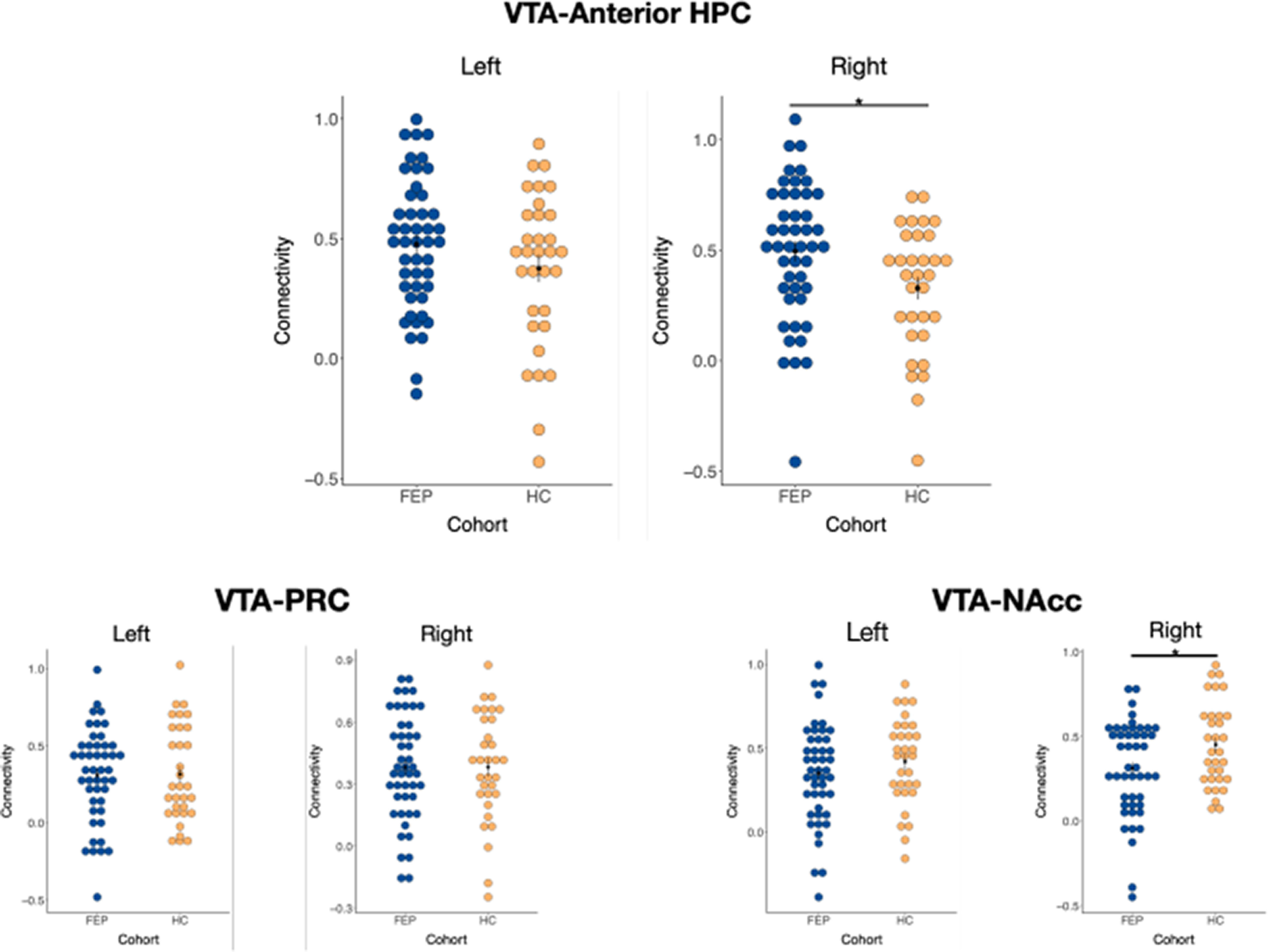
Functional coupling. Axis depicts connectivity of seed to target during resting state fMRI and cohort differences in mean activation. VTA seed to each lateral target: VTA to anterior hippocampus (HPC; top), VTA to PRC (bottom left), and VTA to NAcc (bottom right). FEP cohort depicted in blue and HC cohort depicted in orange. Each dot represents a single participant. (*p < 0.05).

Our analyses indicated that functional coupling between VTA and right anterior hippocampus was significantly greater in FEP versus HC (*t*_(76)_ = 2.40, *p *<* *0.05, Bonferroni corrected, Cohen’s *d *=* *0.56; Fig. 2). Notably, VTA-right anterior hippocampus functional coupling did not differ across medicated and un-medicated FEP individuals. For the right hippocampus, there was no significant difference between individuals on and off medication (*t*_(44)_ = −1.29, *p *>* *0.20). For the right NAcc, there was no significant difference between individuals on and off medication (*t*_(44)_ = −0.23, *p *>* *0.81). A similar pattern to right anterior hippocampus-VTA was observed with left anterior hippocampus-VTA functional coupling, although the result was non-significant (*t*_(76)_ = 1.47, *p *=* *0.15, Bonferroni corrected, Cohen’s *d *=* *0.33; Fig. 2). As a control analysis, we examined functional coupling of the VTA with the PRC, a region also located in the MTL. There were no differences in functional coupling between the VTA and PRC across groups (right PRC: *t*_(76)_ = −0.02 *p *=* *0.98, Cohen’s *d *=* *0.00; left PRC: *t*_(76)_ = −0.08 *p *=* *0.93, Cohen’s *d *= 0.02). Finally, we examined the NAcc, given its role in the mesolimbic circuit, and found that functional coupling between VTA and right NAcc was significantly greater in HC versus FEP (*t*_(76)_ = −1.07, *p *<* *0.05, Cohen’s *d *=* *0.53; Fig. 2). There was no difference between VTA and left NAcc across groups (*t*_(76)_ = −2.27 *p *=* *0.29, Cohen’s *d *=* *0.25). This same pattern of results remained consistent for all the above reported findings when including age and sex as covariates of interest.

Because we observed group differences in VTA-right hippocampus and VTA-right NAcc functional coupling, we then assessed the relationship between these measures and Brief Psychiatric Rating Scale symptom severity scores in the FEP group. We found a trend toward a positive correlation between VTA-right hippocampus functional coupling and overall symptom severity (*r*_(43)_ = 0.24, *p *=* *0.10). This relationship was significant when controlling for age, sex, and antipsychotic status (*r*_(43)_ = 0.30, *p *=* *0.05). These results remained significant when including the VTA-right NAcc into the model (*r*_(39)_ = 0.39, *p* = 0.01) to ensure that findings were specific to the VTA hippocampal circuit. There was no such relationship between VTA-right NAcc (*r*_(43)_ = 0.14, *p *=* *0.36), and no such relationship when including covariates of age, sex, and antipsychotic status (*r*_(43)_ = 0.21, *p *=* *0.18). As well, there were no significant relationships of these neural measures with subscales of positive or negative symptoms (*ps *>* *0.28).

Finally, we conducted a whole brain analysis to see whether there were any significant differences in functional coupling with the VTA outside of ROIs. Notably, a whole-brain analysis within each group showed strong connectivity of the VTA with core regions within the mesolimbic dopamine system, including dorsal striatum, ventral striatum, hippocampus, amygdala, and ventromedial prefrontal cortex (see https://osf.io/dtu5z/ for unthresholded z-maps). Whole-brain analysis did not reveal any significant differences across FEP and HC groups, when using whole brain correction. However, at a more lenient, exploratory threshold (*p* < 0.001, cluster extant > 15), the FEP group showed greater functional coupling of the VTA with the right agranular frontal (BA6), and less functional coupling of the VTA with the right intermediate frontal area (BA8), compared with the CON group. These results should be interpreted with caution given the liberal statistical thresholds.

## Discussion

Results indicated that VTA-hippocampal rsfMRI functional coupling is higher in individuals within their first episode of psychosis in comparison to normative controls. We found that these psychosis-related increases in mesolimbic connectivity were specific to hippocampus/VTA connectivity, as there were no differences in our other MTL ROIs (i.e., PRC), a decrease in functional coupling with the NAcc, and no robust differences in exploratory-whole brain analyses. Further, individual differences in VTA-hippocampus functional coupling within the FEP group correlated to symptom severity as assessed by the Brief Psychiatric Rating Scale.

Recent work has also shown disruptions in mesolimbic-hippocampal circuits across different phases of psychosis. In a sample with chronic psychosis, general psychosis symptoms were correlated with hippocampal-VTA connectivity, although, again, there were no significant group differences in this circuit compared with a control group (Nakamura et al., 2020). Here, we found group differences in VTA-hippocampal connectivity in an FEP sample, which highlights that functional coupling between these regions may distinguish between different phases of psychosis. Differences may have emerged in our study within our FEP group because patients were either drug-naive or only newly exposed to psychosis medication. Increased VTA-hippocampal interactions may be more important in early stages of the disorder, which reflects the lack of group-level differences found in studies of chronic, lasting psychosis treated for long durations with heavy medication. Thus, our data raises the possibility that after prolonged anti-psychotic treatment, individuals with chronic psychosis may more closely resemble controls in regards to mesolimbic circuit function. However, in chronic psychosis, interactions in this circuit may only relate to positive symptoms which may not have been remediated by anti-psychotic medication (Lieberman et al., 2001; Fusar-Poli et al., 2015; Lieberman and First, 2018). Future studies leveraging longitudinal cohorts will provide a better understanding of interactions between mesolimbic-hippocampal functional connectivity, psychosis duration, and medication usage as well as the impact of negative and positive symptoms.

Notably, recent work has demonstrated that mesolimbic-hippocampal interactions differentiate individuals at CHR for psychosis from normative controls. CHR individuals showed reduced hippocampal activity during an event-related novelty/salience task which drives hippocampal-midbrain-striatal response. The authors found that CHR participants had significantly less activation with stimulus novelty in the anterior right hippocampus, but when using functional coupling as an approach to understand the circuit, CHR subjects showed greater VTA-hippocampal connectivity, along with reduced midbrain-striatum connectivity (Modinos et al., 2020). Thus, our findings extend this literature to indicate that patterns of connectivity not only persist into patient’s first episode, but notably also generalize to task-free states. These findings would then support a model by which symptoms associated with mesolimbic-hippocampal interactions, such as delusions and paranoia, may be prominent at baseline and not just during states of novelty. Of note, our study only reports correlation between VTA-hippocampal coupling and symptom severity, and future studies examining this circuit before and after medication administration and the relationship with symptoms will be necessary to support these implications in a broader context.

We also found that functional coupling between the VTA-NAcc circuits was greater in HC versus FEP. Reduced VTA-NAcc connectivity has also been shown during resting state in psychosis-related populations with general severity of symptoms (Nakamura et al., 2020), as well as during salience detection in CHR (Modinos et al., 2020). Connectivity between the VTA and NAcc is known to be critical for initiating and maintaining goal-oriented behavior (Shohamy and Adcock, 2010; Ballard et al., 2011; Berridge, 2012; Wise and Robble, 2020) and alterations in NAcc engagement has been associated with symptoms of anhedonia (Lee et al., 2015; Zhang et al., 2016; Barkus and Badcock, 2019; Borsini et al., 2020), which are prominent in psychosis (Kirkpatrick and Buchanan, 1990; Strauss and Gold, 2012). Together, these findings suggest that psychosis is associated with increased functional coupling between the VTA and hippocampus, and decreased functional coupling between the VTA and striatum.

Critically, this dichotomy between reduced VTA connectivity with the striatum and increased connectivity with the hippocampus could begin to specify the complexity of mesolimbic deficits in psychosis. Namely, psychosis often presents with symptoms that seem to result from hyperdopaminergic activation, such as delusions and paranoia, and symptoms that seem to result from hypodopaminergic activation, such as anhedonia. We propose that divergent patterns across VTA subcircuits could help determine paradoxical nature of symptoms, such that increases in salience detection and delusions occur via hippocampal neuromodulation, whereas decreased goal-motivated behavior occur via striatal neuromodulation. Our analysis of individual differences in symptoms did not show this double-dissociation but rather a positive relationship between hippocampal neuromodulation and overall symptom severity (i.e., positive and negative symptoms). However, engagement of VTA-NAcc circuits has been shown to be sensitive to task context (Murty et al., 2018), and brain-behavior relationships may only emerge in the context of reward. Thus, future studies examining the relationship between VTA subcircuits and positive symptoms during task states will be needed to test our hypotheses.

There are three main limitations to this study we have identified here which can be address in future work. First, while this study sought to describe aspects of psychosis before long duration of heavy medication, many of our FEP participants were not naive to medication. Our current sample did not include enough drug naive individuals to study them in isolation. Second, our only comparison group was a HC group, which did not allow for examination across different neuropsychiatric disorders to determine whether functional alterations of mesolimbic circuits are specific to psychosis. Future studies should investigate how our findings are impacted in comparison to other clinical groups, including early psychosis risk to long-term psychosis patients. Third, as we only evaluated the groups during rest, we cannot conclude any behavioral differences between FEP and CON in this study.

The current study adds to growing literature characterizing neural deficits in FEP. Our findings suggest that the well-documented deficits in hippocampal function and mesolimbic circuits in animal and human findings in psychosis may actually reflect a dysfunction across the two regions, resulting from the hippocampus over-stimulating the VTA, and the VTA releasing excessive amounts of dopamine in the hippocampus. Although our results do not directly indicate overstimulation of VTA or hippocampus independently or directionally, our findings point toward a correlation of VTA-hippocampus disruption in FEP during rest. Critically, these alterations seem to be present, at least, immediately after the onset of the disorder, and are implicative of individual differences in symptom severity of FEP. This characterization provides an important link between animal models of psychosis and human neuroimaging, and serves as a foundation to understand how circuit-level alterations in the mesolimbic system may contribute to the heterogeneity of symptoms in psychosis.

## Synthesis

Reviewing Editor: Ifat Levy, Yale University School of Medicine Decisions are customarily a result of the Reviewing Editor and the peer reviewers coming together and discussing their recommendations until a consensus is reached. When revisions are invited, a fact-based synthesis statement explaining their decision and outlining what is needed to prepare a revision will be listed below. The following reviewer(s) agreed to reveal their identity: Or Duek.

The neural mechanism of psychosis is still unclear. Most studies look into chronic patients; while the contribution of those studies is important, many confounding variables might affect those results. Looking into first-episode psychotic patients, without the effect of chronic medication and hospitalizations, might shed some light on the mechanism of psychosis.

The authors have revised the manuscript according to comments, and have answered all concerns. Some limitations cannot be fully mitigated, but the authors have addressed them sufficiently.

A remaining comment: It was not clear how the authors controlled for age, sex and psychotic status when computing the correlation between symptoms and connectivity (partial correlation? regression?). Please clarify.

Minor issue - MTL appears without expansion (page 3, line 57).

## Author Response

We thank the editor and the reviewers for their thoughtful assessment of the revised

manuscript. We were excited to hear that the manuscript was deemed suitable for

publication. Below, we respond on a point-by-point basis to all remaining concerns. These

revisions have been highlighted in the revised manuscript in blue text. We believe these

edits have resolved any remaining issues.

Comment 1: The species studied is not mentioned in the abstract. Please make sure to

update both the abstract in the article file and on the submission form.

Response: We have now included that this study was performed in male and female human

participants in the abstract (both in the article and submission form).

Comment 2: The authors have revised the manuscript according to comments, and have

answered all concerns. Some limitations cannot be fully mitigated, but the authors have

addressed them sufficiently.

Response: We are grateful that the reviewers agreed that we have addressed the concerns

raised in the prior round of reviews.

Comment 3: It was not clear how the authors controlled for age, sex and psychotic status

when computing the correlation between symptoms and connectivity (partial correlation?

regression?). Please clarify.

Response: We apologize for not clarifying our approach in the methods section. These

variables were controlled for using the ‘partialcorr’ function in Matlab, thus representing a

partial correlation.

Comment 4: MTL appears without expansion (page 3, line 57)

Response: We have corrected this typo, and have now defined our abbreviation of medial

temporal lobe as MTL in its first instantiation.
